# Estimation of utility weights for major liver diseases according to disease severity in Korea

**DOI:** 10.1186/s12876-017-0660-3

**Published:** 2017-09-05

**Authors:** Minsu Ock, So Yun Lim, Hyeon-Jeong Lee, Seon-Ha Kim, Min-Woo Jo

**Affiliations:** 1 Department of Preventive Medicine, Ulsan University Hospital, University of Ulsan College of Medicine, Ulsan, South Korea; 2 Asan Medical Center, Seoul, South Korea; 3 Department of Preventive Medicine, University of Ulsan College of Medicine, 88 Olympic-ro 43-gil, Songpa-Gu, Seoul, 05505 South Korea; 4 Department of Nursing, College of Nursing, Dankook University, Cheonan, South Korea

**Keywords:** Utility weight, Health related quality of life, Hepatitis B, Liver cirrhosis, Hepatocellular carcinoma

## Abstract

**Background:**

The global burden of liver diseases, such as hepatocellular carcinoma and liver cirrhosis, is substantial. In this study, we estimated utility weights of liver disease-related health states in the general population using a visual analogue scale (VAS) and the standard gamble (SG) method.

**Methods:**

Depictions of standardized health states related to major liver diseases were developed based on patient education materials and previous publications. To fully reflect disease progression from diagnosis to prognosis, each health state comprised four parts: diagnosis, symptoms, treatment, and progression and prognosis. A total of 407 participants from the Korean general population evaluated the health states using the VAS and SG methods in computer-assisted personal interviews. After excluding illogical responses, mean utility weights were calculated for each health state.

**Results:**

The utility weights for health states were significantly different according to the existence of inconsistency in general. According to the VAS results, the health state with the highest utility was ‘Chronic hepatitis B virus infection’ (0.64), whereas the health state with the lowest utility was ‘Hepatocellular carcinoma that requires palliative therapy’ (0.17). Similarly, the SG results revealed that the health state with the highest utility was ‘Chronic hepatitis B virus infection’ (0.85), and the health state with the lowest utility was ‘Hepatocellular carcinoma that requires palliative therapy’ (0.40).

**Conclusions:**

The estimated utility weights in this study will be useful to measure the burden of liver diseases and evaluate cost-utility of programs for reducing the burden of liver diseases.

**Electronic supplementary material:**

The online version of this article (10.1186/s12876-017-0660-3) contains supplementary material, which is available to authorized users.

## Background

Burden of disease due to liver diseases, such as hepatocellular carcinoma (HCC) and liver cirrhosis (LC), is substantial, especially in developing countries [1]. More than 1 million people died of liver diseases in 2010 [2]. In particular, among all types of cancers, hepatocellular carcinoma ranked fifth for incidence and second for mortality in developing countries in 2013 [3]. In South Korea (hereinafter Korea), although mortality due to liver diseases was continuously declined, liver diseases were still ranked among the leading causes of death in 2014, both in men (7th) and women (10th) [4].

Liver diseases are chronic illnesses caused by multiple etiological factors, such as virus infection, alcohol consumption, and metabolic disorders, including obesity [5]. Accordingly, patients with liver diseases may experience various symptoms, such as fatigue and depression, and a diverse array of complications, such as ascites and varix bleeding [6, 7]. These symptoms and complications can lead to deterioration in the health-related quality of life. Therefore, management of liver diseases should focus not only on decreasing liver disease-associated mortality, but also on improving health-related quality of life.

Health-related quality of life for various liver disease health states can be expressed as a utility weight, in which 0 indicates being dead and 1 represents full health [8]. Utility weights for health states of certain diseases are useful for representing disease burden with summary measure of population health, such as quality-adjusted life year [9]. Quality-adjusted life year is a generic outcome measure that has been used in cost-utility studies [10]. Utility weights for health states of liver diseases are essential to evaluate the cost-utility of interventions for managing liver diseases.

According to a systematic review article about health-state utility weights in liver diseases, several studies have attempted to measure utility weights for liver disease health states using direct and indirect approaches [11]. However, the majority of studies focused on health states of hepatitis viruses or liver transplant, and few studies have estimated utility weights for health states of HCC. Furthermore, although burden of disease due to liver diseases is comparatively high in Asian countries [1, 12], relatively few studies have been undertaken in Asian countries to measure utility weights for health states of liver diseases. Therefore, in this study, we estimated utility weights for health states of liver diseases, such as HCC, in the general population in Korea.

## Methods

### Participants, survey procedure, and interviewer training

We sampled the general Korean population over 19 years of age, according to age, gender, sub-region (except Jeju island), and education level, to select survey participants using a multistage stratified quota sampling method. We recruited participants in the street in accordance with the pre-determined quotas. The survey was administered by trained interviewers in computer-assisted personal interviews. After consenting to participation in the survey, potential participants responded to screening questions regarding age, gender, and education level. Those who met the criteria for recruitment according to the quotas participated in 2 valuation methods: visual analogue scale (VAS) and the standard gamble (SG). Lastly, selected participants were administered occupation, monthly income, and health condition questions. Specifically, regarding their health condition, participants were asked whether they had an ambulatory care visit in the past 2 weeks, been hospitalized in the past 12 months, or had any diagnosis of disease. Prior to administering the surveys, interviewers were trained using education resources and simulation exercises for approximately 2.5 hours.

### Health states of liver diseases

One of the authors (MO) of the present study drafted descriptions of 8 hypothetical health states of major liver diseases for a general public consumer audience using educational materials designed for patients with liver diseases. The draft health state descriptions were reviewed and modified by one internist who specialized in liver diseases at the Asan Medical Center. The 8 hypothetical health states of liver diseases reflecting disease severity were as follows: chronic hepatitis B virus infection, chronic hepatitis C virus infection, non-alcoholic steatohepatitis, liver cirrhosis, HCC that requires a partial hepatectomy, HCC that requires non-surgical treatment, HCC that requires a liver transplantation, and HCC that requires palliative therapy. Each health state comprised diagnosis, symptoms, treatment, and progress and prognosis, similar to a previous study [13]. Specifically, each health state included details about the diagnosis process; common side effects of treatment, in terms of physical and emotional health; possibility of full recovery; and 5-year survival rate. Explanations of non-surgical treatments of HCC, such as transarterial chemoembolization and radiofrequency ablation, and liver transplantation were also provided. The full descriptions of the 8 health states of liver diseases are available in Additional file 1.

### Valuation method and estimation of utility weight

The preferences of participants for the health states were measured using VAS and SG methods. First, participants assessed 4 of the health states, randomly selected from the 8 health states, and the state of being dead, using the VAS method. For the VAS method, participants were asked to imagine living in the given health state and rate the health state on a scale of 0 (worst imaginable state) to 100 (best imaginable state). If the VAS value for being dead was 0, the utility weight for the health state of a liver disease was calculated as follows: VAS value for the health state/100. If the VAS value for being dead was not 0, the following formula was utilized: (VAS value for the health state – the VAS value for being dead)/(100 – VAS value for being dead).

Next, participants participated in the SG method 5 times for the 5 health states that were also randomly selected from the 8 health states. For the SG method, participants were requested to assess which health state is better (preferable): the given health state of the liver disease or the state of being dead. If participants evaluated that a given health state of liver disease was better than being dead, we asked participants to select their preferred option between two alternatives: 1) live in the given health state or 2) receive treatment with 2 possible outcomes, restoration to full health with probability *p,* or dying immediately with probability one minus *p*. Probability *p* was changed until there was no difference in preference between the 2 alternatives. The probability level started at 50:50 and the interval of minimum probability was 5%. The utility weight for the health state of each liver disease was calculated as follows: *p*/(1- *p*). If participants regarded being dead as preferable to the given health state of liver disease, the utility weight for that response was censored at 0.

### Inconsistency

To determine if participants logically evaluated health states, the number of inconsistencies was determined. An inconsistency was defined as the reverse order of utility weights in health states of liver diseases with known severity levels [14]. Chronic hepatitis B, chronic hepatitis C, and non-alcoholic steatohepatitis were categorized as the least severe group. Liver cirrhosis, HCC that requires a partial hepatectomy, HCC that requires non-surgical treatment, and HCC that requires a liver transplantation were classified as the severe group. HCC that requires palliative therapy was regarded as the most severe group. For example, if utility weights of health states in the most severe group were estimated higher than utility weights of health states in the severe group, we assumed that an inconsistency occurred. Ties between groups were allowed. We counted the number of inconsistencies in both the VAS and SG methods.

### Analysis

The mean utility weights for the health state were calculated according to the existence of inconsistency in each health state. We conducted Chi-square test or Fisher’s exact test to determine the significant difference of inconsistency proportion according to socio-demographic and health condition factors. Furthermore, after excluding values from inconsistent participants, Student’s *t*-test and analysis of variance were applied to identify whether there was significant difference in utility weights depending on the socio-demographic and health condition factors. All statistical analyses were performed using SPSS v20.0 software with a two-sided 5% significance level.

### Ethics approval

This study was approved by the Institutional Review Board of the AMC (S2014-1396-000). Prior to enrollment, we explained the objectives and procedures of this study to the participants and obtained oral informed consent from them.

## Results

A total of 407 participants completed the survey. Table 1 shows the socio-demographic and health condition factors of the participants. Table 2 shows the number of inconsistencies in both the VAS and SG methods. Inconsistent responses were detected from 84 and 125 participants for the VAS and SG methods, respectively (Table 3). Although there were no statistical differences in the inconsistency proportion according to socio-demographic and health condition factors (Table 4), the utility weights for health states were significantly different according to the existence of inconsistency for both the VAS and SG methods in general (Table 3).

**Table 1:** Socio-demographic and health condition factors of participants

Characteristic		N	%
Gender	Male Female	204 203	50.1 49.9
Age group	19-29 30-39 40-49 50-59 60 or more	73 79 86 79 90	17.9 19.4 21.1 19.4 22.1
Educational level	Elementary school or below Middle school High school College or above	14 40 256 97	3.4 9.8 62.7 23.8
Occupation	Manual Non-manual Others	219 78 108	53.7 19.1 26.5
Monthly income	Less than 2.5 million won 2.5-5.0 million won More than 5.0 million won	73 229 105	17.9 56.3 25.8
Ambulatory care visit in past two weeks	Yes No	49 358	12.0 88.0
Hospitalization in past twelve month	Yes No	9 398	2.2 97.8
Morbidity	Yes No	41 366	10.1 89.9
Total		407

**Table 2:** The number of inconsistencies in the visual analogue scale and standard gamble methods

The number of inconsistencies	Visual analogue scale		Standard gamble	
	N	%	N	%
0	323	79.4	282	69.3
1	39	9.6	35	8.6
2	30	7.4	19	4.7
3	10	2.5	21	5.2
4	3	0.7	27	6.6
≥ 5	2	0.5	23	5.6
Total	407	100	407	100

**Table 3:** Utility weights of health states according to consistency

Health states	Mean utility weights of VAS			Mean utility weights of SG		
	Consistent participants	Inconsistent participants	Total	Consistent participants	Inconsistent participants	Total
Chronic hepatitis B virus infection	0.64**	0.44**	0.63	0.85**	0.56**	0.82
Chronic hepatitis C virus infection	0.62**	0.46**	0.60	0.82**	0.53**	0.79
Non-alcoholic steatohepatitis	0.62**	0.43**	0.60	0.85**	0.52**	0.82
Liver cirrhosis	0.35*	0.43*	0.35	0.57	0.50	0.56
HCC that requires a partial hepatectomy	0.36	0.37	0.37	0.62*	0.53*	0.61
HCC that requires non-surgical treatments	0.29*	0.35*	0.29	0.55	0.53	0.54
HCC that requires a liver transplantation	0.45**	0.53**	0.47	0.67**	0.50**	0.67
HCC that requires palliative therapy	0.17**	0.29**	0.16	0.40**	0.57**	0.39

*VAS* visual analogue scale, *SG* standard gamble, *HCC* hepatocellular carcinoma

*P-value < 0.05; **P-value < 0.01

**Table 4:** Inconsistency proportion according to socio-demographic and health condition factors

		Visual analogue scale				Standard gamble			
		Consistent participants		Inconsistent participants		Consistent participants		Inconsistent participants	
		N	%	N	%	N	%	N	%
Gender	Male	165	80.9	39	19.1	144	70.6	60	29.4
	Female	158	77.8	45	22.2	138	68.0	65	32.0
Age (year)	~49	181	76.1	57	23.9	166	69.7	72	30.3
	50~	142	84.0	27	16.0	116	68.6	53	31.4
Education level	High school or below	244	78.7	66	21.3	214	69.0	96	31.0
	College or above	79	81.4	18	18.6	68	70.1	29	29.9
Occupation	Manual	169	77.2	50	22.8	149	68.0	70	32.0
	Non-manual	64	82.1	14	17.9	51	65.4	27	34.6
	Others	88	81.5	20	18.5	80	74.1	28	25.9
Monthly income	Less than 2.5 million won	58	79.5	15	20.5	57	78.1	16	21.9
	2.5-5.0 million won	177	77.3	52	22.7	151	65.9	78	34.1
	More than 5.0 million won	88	83.8	17	16.2	74	70.5	31	29.5
Ambulatory care visit in past 2 weeks	Yes	41	83.7	8	16.3	35	71.4	14	28.6
	No	282	78.8	76	21.2	247	69.0	111	31.0
Hospitalization in past 12 months	Yes	7	77.8	2	22.2	4	44.4	5	55.6
	No	316	79.4	82	20.6	278	69.8	120	30.2
Morbidity	Yes	37	90.2	4	9.8	29	70.7	12	29.3
	No	286	78.1	80	21.9	253	69.1	113	30.9

After excluding values from inconsistent participants, utility weights from the SG method were higher than those from the VAS method. The highest VAS utility weight was chronic hepatitis B virus infection (0.64), followed by non-alcoholic steatohepatitis (0.618) and chronic hepatitis C virus infection (0.616). The lowest VAS utility weight was HCC that requires palliative therapy (0.17), followed by HCC that requires non-surgical treatments (0.29), and liver cirrhosis (0.35). The highest SG utility weight was non-alcoholic steatohepatitis (0.855), followed by chronic hepatitis B virus infection (0.848) and chronic hepatitis C virus infection (0.82). The lowest SG utility weight was HCC that requires palliative therapy (0.40), followed by HCC that requires non-surgical treatments (0.55) and liver cirrhosis (0.57). In general, the mean utility weights did not differ according to socio-demographic and health condition factors (Table 5).

**Table 5:** Utility weights according to socio-demographic and health condition factors

		Chronic hepatitis B virus infection		Chronic hepatitis C virus infection		Non-alcoholic steatohepatitis		Liver cirrhosis		HCC that requires a partial hepatectomy		HCC that requires non-surgical treatments		HCC that requires a liver transplantation		HCC that requires palliative therapy	
		VAS	SG	VAS	SG	VAS	SG	VAS	SG	VAS	SG	VAS	SG	VAS	SG	VAS	SG
Gender	Man	0.60	0.77	0.58	0.74	0.58	0.77	0.36	0.55	0.35	0.60	0.30	0.51	0.47	0.63	0.19	0.44
	Woman	0.59	0.75	0.59	0.73	0.58	0.73	0.38	0.56	0.37	0.59	0.29	0.57	0.47	0.59	0.20	0.46
Age group	19-29	0.60	0.78	0.59	0.75	0.56	0.78	0.38	0.50	0.37	0.57	0.30	0.51	0.51	0.60	0.20	0.44
	30-39	0.56	0.82	0.59	0.81*	0.61	0.82	0.36	0.59	0.33	0.64	0.30	0.54	0.48	0.67	0.17	0.43
	40-49	0.58	0.66	0.63	0.62*	0.59	0.65	0.44**	0.52	0.40	0.54	0.34	0.55	0.54**	0.54	0.25	0.44
	50-59	0.57	0.76	0.59	0.69	0.51	0.77	0.35	0.50	0.32	0.59	0.27	0.49	0.43	0.62	0.19	0.44
	≥60	0.67	0.78	0.55	0.79	0.61	0.75	0.29**	0.63	0.38	0.65	0.29	0.60	0.41**	0.64	0.18	0.51
Education level	High school or below	0.60	0.75	0.59	0.73	0.59	0.75	0.37	0.54	0.36	0.59	0.30	0.54	0.47	0.31	0.19	0.45
	College or above	0.58	0.81	0.59	0.74	0.54	0.77	0.36	0.59	0.34	0.62	0.29	0.54	0.47	0.29	0.20	0.45
Occupation	Manual	0.60	0.75	0.58	0.71	0.58	0.74	0.35	0.52	0.35	0.58	0.31	0.53	0.48	0.59	0.19	0.43
	Non-manual	0.58	0.76	0.59	0.72	0.57	0.75	0.39	0.59	0.35	0.58	0.27	0.54	0.52	0.67	0.21	0.47
	Others	0.61	0.80	0.59	0.77	0.58	0.78	0.38	0.58	0.39	0.65	0.31	0.57	0.43	0.63	0.19	0.48
Monthly income	Less than 2.5 million won	0.67*	0.77	0.55	0.72	0.60	0.75	0.32	0.55	0.37	0.61	0.29	0.52	0.43	0.63	0.19	0.42
	2.5-5.0 million won	0.56*	0.76	0.60	0.72	0.56	0.76	0.37	0.56	0.36	0.60	0.29	0.56	0.48	0.62	0.18	0.45
	More than 5.0 million won	0.62	0.76	0.59	0.76	0.59	0.74	0.38	0.54	0.35	0.57	0.31	0.53	0.47	0.59	0.23	0.48
Ambulatory care visit in past 2 weeks	Yes	0.54	0.75	0.52*	0.69	0.60	0.76	0.31	0.54	0.34	0.63	0.29	0.58	0.39	0.66	0.18	0.45
	No	0.60	0.76	0.60*	0.74	0.57	0.75	0.37	0.55	0.36	0.59	0.30	0.54	0.48	0.61	0.20	0.45
Hospitalization in past 12 months	Yes	0.54	0.55	0.53	0.29	0.81	0.62	0.34	0.38	0.43	0.47	0.35	0.78	0.42	0.49	0.19	0.47
	No	0.60	0.77	0.59	0.74	0.57	0.76	0.37	0.56	0.36	0.60	0.30	0.54	0.47	0.62	0.19	0.45
Morbidity	Yes	0.67	0.79	0.57	0.70	0.56	0.80	0.35	0.61	0.37	0.59	0.30	0.54	0.43	0.63	0.17	0.45
	No	0.59	0.76	0.59	0.73	0.58	0.75	0.37	0.54	0.36	0.60	0.30	0.54	0.47	0.61	0.20	0.45

*VAS* visual analogue scale, *SG* standard gamble, *HBV* hepatitis B virus, *HCV* hepatitis C virus, *HCC* hepatocellular carcinoma

*P-value < 0.05; **P-value < 0.01

## Discussion

In this study, the utility weights of various health states related to major liver diseases, such as LC and HCC, were estimated by using the VAS and SG methods among 407 participants recruited from the general population of Korea. The strength of this study is that we calculated utility weights in East Asia for major liver diseases. In addition, to the best of our knowledge, it is rare that the general population is the target group among studies evaluating utility weights of liver diseases. Although there has been debate regarding the appropriate target group for utility weight study, it is recommended to use utility weights from the general population, if possible [15, 16]. Therefore, it is meaningful that the assessment of preferences by major liver diseases in the general population was confirmed in this study.

The estimates of the utility weight of liver diseases can vary depending on the type of participant, such as patients, the general population, or healthcare professionals, and cultural differences [17]. Most of the previous studies investigating utility weights of liver diseases targeted patients and expert panels [11]. Levy AR, et al. conducted a multinational survey (United States, Canada, United Kingdom, Spain, Hong Kong, and mainland China) to estimate utility weights from hepatitis B virus infected and uninfected persons [12]. They reported that SG utility weights of 0.77 for chronic hepatitis B, 0.80 for compensated cirrhosis, 0.35 for decompensated cirrhosis, 0.41 for HCC, 0.65 for liver transplant (1st year), and 0.76 for liver transplant (> 1st year). Furthermore, utility weights from mainland China and Hong Kong were lower than those from other countries. Although the comparability was limited due to difference in classification of liver diseases and standardized descriptions, we believe the results from Levy AR et al. were similar to ours. However, as Levy AR et al. pointed out, the utility weights of liver diseases are likely to be significantly different among countries; more country-specific utility weights need to be estimated and compared.

The methods of measuring utility weights are divided into 2 categories: indirect method using questionnaire, such as EQ-5D, and direct method using VAS, time trade-off, or SG [11]. In this study, we selected the VAS and SG methods. The VAS is usually used to familiarize participants with health states; therefore, we also used the VAS method prior to the SG method. The SG measures the preference of respondents in uncertainty and thus has the advantage of being directly based on the expected utility theory of von Neumann-Morgenstern [18]. Accordingly, the SG method has been regarded as a gold standard in utility studies [19]. However, probability weighting and loss aversion have been identified as limitations of the SG method [20]. Furthermore, the SG method might be relatively more difficult to understand for the general public than other valuation methods, such as the VAS. In this study, inconsistent responses were detected at a higher rate in the SG than VAS method. These results empirically supported the idea that the public has more difficulty understanding the SG method. Therefore, it may be meaningful to compare and evaluate the utility weights from multiple valuation methods, rather than depending on a single valuation method. Furthermore, when the SG method is used, it is necessary to help respondents understand the SG, and use the responses of participants who demonstrate a strong understanding of the SG.

As we expected, preferences from the VAS method were lower than from the SG method for all health states [13]. In addition, we expected that the utility weights of health states would decrease as the severity of liver diseases increases; our expectations were confirmed in this study. For example, the SG utility weights were 0.62, 0.55, and 0.40 for HCC that requires a partial hepatectomy, HCC that requires non-surgical treatment, and HCC that requires palliative therapy, respectively. The health state of HCC that requires a liver transplantation was rated higher than other health states, except for the health states of chronic hepatitis B virus infection, chronic hepatitis C virus infection, and non-alcoholic steatohepatitis, which were without mortality information. Furthermore, the variation of utility weights according to the severity of liver diseases was more prominent in consistent participants than in inconsistent participants. Therefore, we believe that using values from only the consistent participants is a more logical approach in a utility weight study.

This study has the following limitations. First, the name of the liver diseases may have influenced the evaluation of preference in the general population. For the convenience of the survey, the names of the liver diseases were exposed to participants. Therefore, preferences of participants might have been influenced by preconceived ideas based on the name of the liver disease, rather than the overall aspect of the health states as described in the study. Second, in this study, we did not evaluate all 8 health states, in order to reduce the cognitive burden on participants. This suggests that the differences in utility weights among health states may be underestimated. If participants assessed the utility weights of all 8 health states, the variance of utility weights would be more significant.

## Conclusions

In this study, we estimated the utility weights of 8 health states related to liver diseases, such as LC and HCC, using the VAS and SG methods, in the general population of Korea. The estimated utility weights can be used to measure the burden of liver diseases using a summary measure of population health, such as quality-adjusted life year and quality-adjusted life expectancy. Moreover, the results from this study can be useful to evaluate the cost-utility of vaccination programs for hepatitis virus and HCC screening programs.

## Additional file

Additional file 1: Standardized health states related to major liver diseases. The full descriptions of the 8 health states of liver diseases are available. (DOCX 22 kb)

## Abbreviations

HCC: Hepatocellular carcinoma
LC: Liver cirrhosis
VAS: visual analogue scale
SG: standard gamble
