# Supplementary material for: Estimation of utility weights for major liver diseases according to disease severity in Korea
Source: BMC Gastroenterol. 2017 Sep 5;17:103. doi: 10.1186/s12876-017-0660-3 (PMC5584479; doi:10.1186/s12876-017-0660-3)
Supplement: Additional file 1: — Standardized health states related to major liver diseases. The full descriptions of the 8 health states of liver diseases are available. (DOCX 22 kb) [file 12876_2017_660_MOESM1_ESM.docx]

**Additional file 1: Standardized health states related to major liver diseases**

The following are techniques for diseases associated with hepatitis virus and hepatocellular carcinoma. First, carefully read the various treatments described below.

⦁ Trans-arterial chemoembolization: This method is a kind of non-surgical treatment of hepatocellular carcinoma, causing necrosis of the liver locally. Patients are hospitalized and fast beginning the day before surgery. Local anesthesia is done before the surgery; a painkiller and sedative are used. A thin tube of approximately 2 - 3mm width is inserted through the femoral artery in the groin and directed to the hepatic artery. A vascular contrast media is injected to obtain information, such as the location of the liver size and blood supply aspects. Then, the anticancer drug is injected into the site of the hepatocellular carcinoma; the drug will block the flow of blood to the site. During the procedure, you may experience nausea, vomiting, abdominal pain, and right shoulder pain. Absolute rest is required for a certain period of time after the treatment and the effects of treatment are assessed through computer tomography afterwards to determine if the treatment was successful. Of treated patients, 5% may present with complications such as bleeding, infection, abscess, adjacent organ damage, and pneumothorax.

⦁ Percutaneous ethanol injection therapy: This method is a kind of non-surgical treatment for hepatocellular carcinoma, causing necrosis of the liver locally. Patients are hospitalized and begin fasting the day before surgery. Local anesthesia is performed before the surgery; a painkiller and sedative are used. The procedure is performed under ultrasonographic guidance. A needle is inserted into the hepatocellular carcinoma region, and ethanol is injected. During the procedure, you may experience nausea, vomiting, abdominal pain, and right shoulder pain. Absolute rest is required for a certain period of time after the treatment and the effects of treatment are assessed through computer tomography afterwards to determine if the treatment was successful. Of treated patients, 5% may present with complications such as bleeding, infection, abscess, adjacent organ damage, and pneumothorax.

⦁ Radiofrequency ablation: This method is a kind of non-surgical treatment of hepatocellular carcinoma, causing necrosis of the liver locally. Patients are hospitalized and begin fasting the day before surgery. Local anesthesia is performed before surgery; a painkiller and sedative are used. The procedure is performed under ultrasonographic guidance. A needle is inserted into the liver region, and generates a high frequency that burns the liver. During the procedure, you may experience nausea, vomiting, abdominal pain, and right shoulder pain. Absolute rest is required for a certain period of time after the treatment and the effects of treatment are assessed through computer tomography afterwards to determine if the treatment was successful. Of treated patients, 5% may present with complications such as bleeding, infection, abscess, adjacent organ damage, and pneumothorax.

⦁ Liver transplantation: The surgery is performed to replace a diseased liver, such as hepatocellular carcinoma and severe liver cirrhosis, with a healthy liver from donors. Patients are hospitalized 3-7 days before surgery, and fasted from the day before surgery. Surgery is done for about 15-20 hours under general anesthesia. After surgery and 3-4 days in the intensive care unit treatment, patients are in sterile ward in one to two weeks and then moved to the normal ward. The length of stay is up to four months and at least 4 weeks. The mortality within four months after the liver transplantation is 5–10%. Liver transplantation can cause complications such as rejection reaction, bleeding, infection, blood vessel constriction, and blood clots, bile leakage and stenosis and kidney failure, and also you may need a lot of blood transfused.

1) Chronic hepatitis B virus infection

- Diagnosis: You underwent a blood test such as liver function tests and ultrasound of the abdomen recently. After 1-2 weeks, abdominal ultrasound is normal, but you were diagnosed with chronic hepatitis B status.

- Symptoms: There are no particular symptoms but can be easily tired or have no appetite. If symptoms are severe, nausea, vomiting, muscle aches and low grade fever may present, and urine color may become darken, or skin and eyes may turn yellow.

- Treatment: If liver function index is not high, it is observed without any treatment, but if the liver function index is high, you generally receive medications for 1 year or more. Due to medication, you may present with flu-like symptoms, such as headache (flu-like symptom), and can have side effects such as decreased renal function. Be sure to visit the hospital regularly during treatment; you will undergo blood tests including liver function tests and abdominal ultrasound scan at intervals of 3-6 months.

- Disease course and prognosis: You are worried that the virus infection could progress to cirrhosis or cancer. It proceeds to cirrhosis at a rate of 5% and to hepatocellular carcinoma at a rate of approximately 1% per year.

2) Chronic hepatitis C virus infection

- Diagnosis: You underwent a blood test, such as liver function tests, or an ultrasound of the abdomen recently. After 1-2 weeks, you find out that the abdominal ultrasound is normal, but you were diagnosed with chronic hepatitis C status.

- Symptoms: There are no particular symptoms, but you can easily tire or have no appetite. If symptoms are severe, nausea, vomiting, muscle aches, and low grade fever may be present, and urine color may become darken, or skin and eyes may turn yellow.

- Treatment: You can try the medication. Due to medication, you may present with flu-like symptoms, such as headache (flu-like symptom), and can have side effects such as anemia and leukopenia. Be sure to visit the hospital regularly during treatment; you will undergo blood tests, including liver function tests and abdominal ultrasound scans, at intervals of 3-6 months.

- Disease course and prognosis: You are worried that the virus infection could progress to cirrhosis or cancer. In approximately 5-20% of cases, it will proceed to cirrhosis after 20-25 years.

3) Non-alcoholic steatohepatitis

- Diagnostics: You underwent a blood test, such as liver function tests, or an ultrasound of the abdomen recently. As a result, you were diagnosed with nonalcoholic steatohepatitis.

- Symptoms: There are no particular symptoms but you may experience fatigue.

- Treatment: Drug therapy is not effective yet; lifestyle improvements are required. Lose weight, limit fat intake and smoking, practice abstinence, and continue to exercise. You can try your medication. However, you should regularly visit hospitals and clinics, and you will undergo a blood test, such as liver function tests, and ultrasound of the abdomen at 3-6 months intervals.

- Disease course and prognosis: You are a little worried that nonalcoholic steatohepatitis could progress to another disease. It proceeds to cirrhosis in 10 to 20% of cases.

4) Liver cirrhosis

- Diagnosis: You underwent a blood test, such as liver function tests, or an ultrasound of the abdomen recently. The result was diagnosis of liver cirrhosis.

- Symptoms: A wide variety of symptoms exist. You become easily tired, have no appetite, and lose weight. The color of urine darkens, the skin or eyes may turn yellow, and you have itching. If symptoms are severe, ascites, edema of both legs, hematemesis due to esophageal variceal bleeding and hematochezia may develop. There can also be infection of ascites and you may lose consciousness or have personality changes due to hepatic coma.

- Treatment: Lifestyle improvement is needed first. Consume adequate calories and protein, and follow a low-salt diet; quit smoking and drinking. Receive medical treatment or undergo surgery depending on symptoms. If ascites occurs, use diuretics or undergo paracentesis to remove the fluid. If infection occurs in ascites, use antibiotics. If there is esophageal variceal bleeding, you will receive medical treatment and undergo endoscopic procedures. If the liver cirrhosis is severe, there is no effective treatment other than liver transplant. You should regularly visit hospitals and clinics, and you will undergo blood tests, such as liver function tests and ultrasounds of the abdomen at 3-6 months intervals.

- Disease course and prognosis: You may develop fear of death or depression that the liver cirrhosis could progress to hepatocellular carcinoma or you may die due to cirrhosis. In the case of severe liver cirrhosis, your daily life activities are limited. In the case of severe cirrhosis, 75% of patients die from complications within 5 years.

5) Hepatocellular carcinoma that requires a partial hepatectomy

- Diagnosis: You underwent a blood test, such as liver function tests, or an ultrasound of the abdomen recently. As a result, partial liver resection was diagnosed with hepatocellular carcinoma.

- Symptoms: Generally there are no special symptoms. A lump may be palpable on the right upper quadrant of the abdomen and it can be painful. In addition, the color of urine darkens and the skin or eyes may turn yellow. You may have no appetite and lose weight.

- Treatment: You need to undergo partial hepatectomy. The surgery is under general anesthesia. The surgery can be laparoscopic surgery or open surgery, and the hospital stay is 14 days. There may be a risk of complications from the surgical procedure, such as infection, organ damage, and bleeding. A complete resection is confirmed by pathologic examination 1-2 weeks after surgery. You need to visit the hospital regularly during the 3-6 months after the initial surgery, and increase the visit interval after that period.

- Disease course and prognosis: In the case of laparoscopic surgery, about 2.5 cm wounds are left on the three to four incisions and open surgery will leave a wound in the abdomen that is about 15 cm. The fear of cancer recurrence and death due to cancer may develop, and you are more likely to be depressed. Some may experience problems such as liver dysfunction, sepsis after liver resection. Prognosis is different depending on the type of hepatocellular carcinoma and pre-operative state; your 5-year survival rate is about 50%.

6) Hepatocellular carcinoma that requires non-surgical treatment

- Diagnosis: You underwent a blood test, such as liver function tests, or an ultrasound of the abdomen recently. As a result, you were diagnosed with hepatocellular carcinoma receiving nonsurgical treatment.

- Symptoms: Generally, there are no special symptoms. A lump may be palpable on the right upper quadrant of the abdomen and it can be painful. In addition, the color of urine darkens and the skin or eyes may turn yellow. You may have no appetite and lose weight.

- Treatment: Because the surgical resection is not possible, you receive non-surgical treatment that induces focal necrosis of the liver via the skin. These non-surgical treatments include transarterial chemoembolization, percutaneous ethanol injection, and radiofrequency ablation using high frequency. The procedure is done under local anesthesia or general anesthesia depending on the procedure, and the hospital stay is from at least 3 days to a maximum of 2 weeks, but you can be hospitalized several times. This procedure can cause complications such as bleeding, infection, abscess, adjacent organ damage, and pneumothorax in approximately 5% of cases. You need to visit the hospital regularly during the 3-6 months after the initial surgery, and increase the visit interval after that period.

- Disease course and prognosis: The fear of cancer recurrence and death due to cancer may develop, and you are more likely to be depressed. Prognosis is different depending on the type of hepatocellular carcinoma and pre-operative state; your 5-year survival rate is about 30%.

7) Hepatocellular carcinoma that requires a liver transplantation

- Diagnosis: You underwent a blood test, such as liver function tests, or an ultrasound of the abdomen recently. As a result, you were diagnosed with hepatocellular carcinoma that requires a liver transplantation.

- Symptoms: You may not have any symptoms, but a wide variety of symptoms similar to liver cirrhosis usually occur. A lump may be palpable on the right upper quadrant of the abdomen and it can be painful. You become easily tired, have no appetite, and lose weight. The color of urine darkens, the skin or eyes may turn yellow, and you have itching. If symptoms are severe, ascites, edema of both legs, hematemesis due to esophageal variceal bleeding, and hematochezia may develop. There also can be infection of ascites and you may lose consciousness or have personality changes due to hepatic coma.

- Treatment: You must undergo liver transplantation because surgical resection is not possible. All of the original liver is removed and the liver of a healthy donor is transplanted through a liver transplantation. Liver transplantation is done under general anesthesia; the hospitalization period is a minimum of 4 weeks, up to as long as 4 months. The mortality within 4 months of liver transplantation is 5-10%. Liver transplantation can cause complications such as rejection reaction, bleeding, infection, blood vessel constriction, thrombosis, bile leakage and stenosis, and kidney failure, and you may need a lot of blood transfused. You undergo outpatient treatment every 1-2 weeks during the initial 3 months after surgery and if you reach a stable state, you visit an outpatient clinic every 2 months. You must continue taking immunosuppressive drugs. Due to the immunosuppressive drugs, you may experience side effects such as headaches, nausea, high blood pressure, and kidney function decline.

- Disease course and prognosis: The fear of cancer recurrence and death due to cancer may develop, and you experience the extreme fear of a surgery as major as liver transplantation. You are more likely depressed. The surgery will leave a wound in the abdomen that is about 20 cm. Prognosis is different depending on the type of hepatocellular carcinoma and pre-operative state; a 5-year survival rate after a successful liver transplant is about 80%.

8) Hepatocellular carcinoma that requires palliative therapy

- Diagnosis: You underwent a blood test, such as liver function tests, or an ultrasound of the abdomen recently. As a result, you were diagnosed with hepatocellular carcinoma that requires palliative therapy.

- Symptoms: You may not have any symptoms, but a wide variety of symptoms similar to liver cirrhosis usually occur. A lump may be palpable on the right upper quadrant of the abdomen and it can be painful. You become easily tired, have no appetite, and lose weight. The color of urine darkens, the skin or eyes may turn to yellow, and you have itching. If symptoms are severe, ascites, edema of both legs, hematemesis due to esophageal variceal bleeding, and hematochezia may develop. There also can be infection of ascites and you may lose consciousness or have personality changes due to hepatic coma.

- Treatment: You do not receive the treatment of hepatocellular carcinoma and liver transplantation because of cancer progression and only receive palliative therapy. You may undergo chemotherapy, but only about 10% of patients respond to treatment. If ascites occurs, diuretics are used or paracentesis is performed to remove the fluid. If infection occurs in ascites, antibiotics are used. If esophageal variceal bleeding is present, medical treatment is administered and endoscopic procedures are performed.

- Disease course and prognosis: The fear of death due to cancer may develop, and you are more likely to be depressed. Daily life can be difficult, depending on the progress of the cancer. A 5-year survival rate is less than 5%.
